# Investigation of the effect of debridement combined with antibiotic-loaded bone cement on pain and psychological status in diabetic foot ulcer patients

**DOI:** 10.3389/fmed.2025.1652992

**Published:** 2025-10-15

**Authors:** Ke Yang, Yijun Huang, Yinxu Zhang, Yin Wen, Linjie Yan, Wei Chen, Zairong Wei, Kaiyu Nie

**Affiliations:** ^1^Department of Burns and Plastic Surgery, Affiliated Hospital of Zunyi Medical University, Zunyi, Guizhou, China; ^2^The Collaborative Innovation Center of Tissue Damage Repair and Regeneration Medicine of Zunyi Medical University, Zunyi, Guizhou, China

**Keywords:** diabetic foot ulcer, pain level, psychological status, antibiotic-loaded bone cement, debridement

## Abstract

**Objective:**

This study aimed to investigate the changes in pain levels and psychological status in patients with diabetic foot ulcers (DFUs) after accepting the debridement combined with antibiotic-loaded bone cement (ALBC), which offers insight into a comprehensive treatment including pain management and psychological intervention with DFUs.

**Methods:**

We conducted a retrospective analysis of 103 patients with DFUs meeting the inclusion criteria at a tertiary academic hospital, divided into pain group (*n* = 61) and numbness group (*n* = 42), anxiety group (*n* = 67) and non-anxiety group (*n* = 36), and depression group (*n* = 16) and non-depression group (*n* = 87). All patients were assessed pain levels and psychological status with brief pain inventory (BPI) and hospital anxiety depression scale (HADS) before and after debridement combined with ALBC.

**Results:**

The primary outcomes were that pain degree score, pain-related impact score, and anxiety score of the depression group were higher than those of the non-depression group (*p* < 0.001). The total scores of pain severity and pain-related effects in the pain group decreased after debridement combined with ALBC (*p* = 0.001, *p* < 0.001), but these scores were always higher than those of the no-pain group (*p* < 0.001). Moreover, the anxiety and depression scores also decreased in most patients who also had a good wound-healing process (*p* < 0.001).

**Conclusion:**

These findings suggest that debridement combined with ALBC of DFU patients may be associated with alleviation of pain and improvement in psychological status in various aspects including controlling infection, promoting wound healing, and reducing frequent treatment, as a recommendation for the widespread therapy used in the clinical treatment of DFUs.

## 1 Introduction

Diabetic foot ulcers (DFUs) are among the most prevalent and severe chronic complications of diabetes, characterized by a high disability rate and substantial social and economic burden. It is studied that over 550 million people worldwide suffer from diabetes, with approximately 18.6 million individuals developing foot ulcers each year (1). The amputation rate for DFUs can reach 20%, and the five-year mortality rate hovers around 30%. In the United States, the annual cost of treating DFUs is estimated to range from $9 billion to $13 billion (2). The development of DFUs is primarily linked to distal neuropathy in the lower extremities and varying degrees of vascular damage in diabetic patients, which leads to foot infections, ulcers, and/or deep tissue damage. The main clinical manifestations include pain, numbness, intermittent claudication, and ischemic necrosis of the foot. Notably, pain is the central clinical symptom for DFU patients, which not only affects their physical health but also frequently leads to psychological distress. A meta-analysis revealed that 47% of DFU patients had experienced depressive symptoms (3). This negative emotional state may induce fluctuations in blood glucose levels through the hypothalamic–pituitary–adrenal axis, creating a “metabolic-psychological” vicious cycle that ultimately delays wound healing and worsens prognosis (4). Consequently, addressing multimodality treatment on the DFU patients like pain management and psychological intervention is becoming a critical component of their clinical management (5).

Antibiotic-loaded bone cement (ALBC) refers to bone cement, primarily composed of polymethylmethacrylate (PMMA), which is evenly impregnated with antibiotics (6). It is extensively utilized in orthopedic surgery as an effective tool for preventing and treating bone and joint infections, commonly used in joint replacement surgeries and fracture fixation procedures (7). The main function of ALBC is to fill bone defects to stabilize the prosthesis or fracture site, while simultaneously releasing high-concentration antibiotics locally through diffusion, which can effectively prevent postoperative infections and reduce the need for long-term intravenous antibiotics, thereby minimizing the burden on the liver and kidneys.

Current studies in the treatment of DFUs have focused on controlling infection and promoting ulcer healing. One promising approach is the use of debridement combined with ALBC, which can gradually release high concentrations of antibiotics locally for 4 to 6 weeks after the removal of necrotic tissue and the preservation of healthy tissue, significantly enhancing its anti-infective efficacy (8). In addition, the microporous structure of ALBC not only provides a three-dimensional scaffold for granulation tissue proliferation but also induces membrane formation, thereby promoting wound repair (9). For example, a meta-analysis by Chen et al. demonstrated that ALBC treatment could significantly reduce the wound healing time and the frequency of debridement without increasing the incidence of complications (10). Therefore, we speculate that debridement combined with ALBC not only effectively inhibits the infection of DFUs but also accelerates ulcer healing, which may further help alleviate patient pain and improve their psychological well-being. However, there is currently a lack of sufficient evidence-based medical data to support these claims. Considering this, this study aims to investigate the changes in pain levels and psychological status of DFU patients in the Qianbei region of southwest China before and after the treatment of debridement combined with ALBC, hoping to provide a theoretical basis for establishing an integrated model of pain management and psychological intervention in DFU treatment using debridement combined with ALBC.

## 2 Materials and methods

### 2.1 Study participants

The study included 198 patients diagnosed with DFUs regardless of the pain status at the Affiliated Hospital of Zunyi Medical University from January 2022 to July 2024. Inclusion criteria: patients all aged over 18 years; patients with a verified diagnosis of DFUs defined as Wagner grade 2 or above; patients willing to accept debridement combined with ALBC treatment; and patients with the ability to write and read Chinese fluently. Exclusion criteria: patients with non-diabetic foot ulcers, other serious complications, malignancies, severe heart, liver, or kidney dysfunction, mental illness, cognitive and communication disorders, and interrupted follow-up. After excluding 95 patients, a total of 103 DFU patients were ultimately enrolled. This study was reviewed and approved by the Medical Ethics Committee of the Affiliated Hospital of Zunyi Medical University (No. KLL-2024-697). Written informed consent was deemed unnecessary due to the study’s retrospective design.

### 2.2 Clinical indicators

Patient medical history and data related to questionnaires were collected. The contents included: (1) demographic data, such as age, gender, body mass index (BMI), etc.; (2) disease-related information, such as duration of diabetes, Wagner classification, pain assessment, psychological assessment, etc.; and (3) laboratory indicators, such as white blood cell count, hemoglobin, albumin, and fasting blood glucose levels, etc.

### 2.3 Clinical scale

#### 2.3.1 Brief pain inventory

The BPI scale, a reliable and effective tool for assessing pain levels in patients, was developed by the Pain Research Group in the Department of Neurology at the University of Wisconsin. The BPI includes 4 items to assess pain severity and 7 items to assess the impact of pain on daily life, work, emotions, etc. Each item is scored on a scale from 0 to 10, with pain severity ranging from 0 (no pain) to 10 (worst possible pain), and the pain-related impact ranging from 0 (no effect) to 10 (complete effect) (11). To evaluate the degree of pain and its impact on quality of life, the Chinese version of the BPI was used in this study. Assessments were conducted in a separate room for privacy and confidentiality purposes.

#### 2.3.2 Hospital anxiety depression scale

The HADS scale, developed by Zigmond AS and Snaith RP in 1983, is mainly used to assess and quantify the anxiety and depression of patients. The HADS consists of two subscales: HADS-A (anxiety) and HADS-D (depression), with 7 items each. Each item is scored from 0 to 3, and the total score of each subscale ranges from 0 to 21. The total score of 0–7 is usually regarded as the normal range; 8–10 may indicate mild anxiety or depression; 11–14 may indicate moderate anxiety or depression; and 15–21 points may indicate severe anxiety or depression (12). HADS has been validated and is one of the tools recommended by the National Institute for Health and Care Excellence for diagnosing depression and anxiety, and Wu et al. (13) had found a HADS-D threshold of 7 or higher has been shown to optimize the combined sensitivity and specificity when screening for major depression. In this study, the Chinese version of HADS was used to assess and quantify patients’ anxiety and depression levels. Assessments were conducted in a separate room for privacy and confidentiality purposes.

### 2.4 Study design

**Figure 1:**
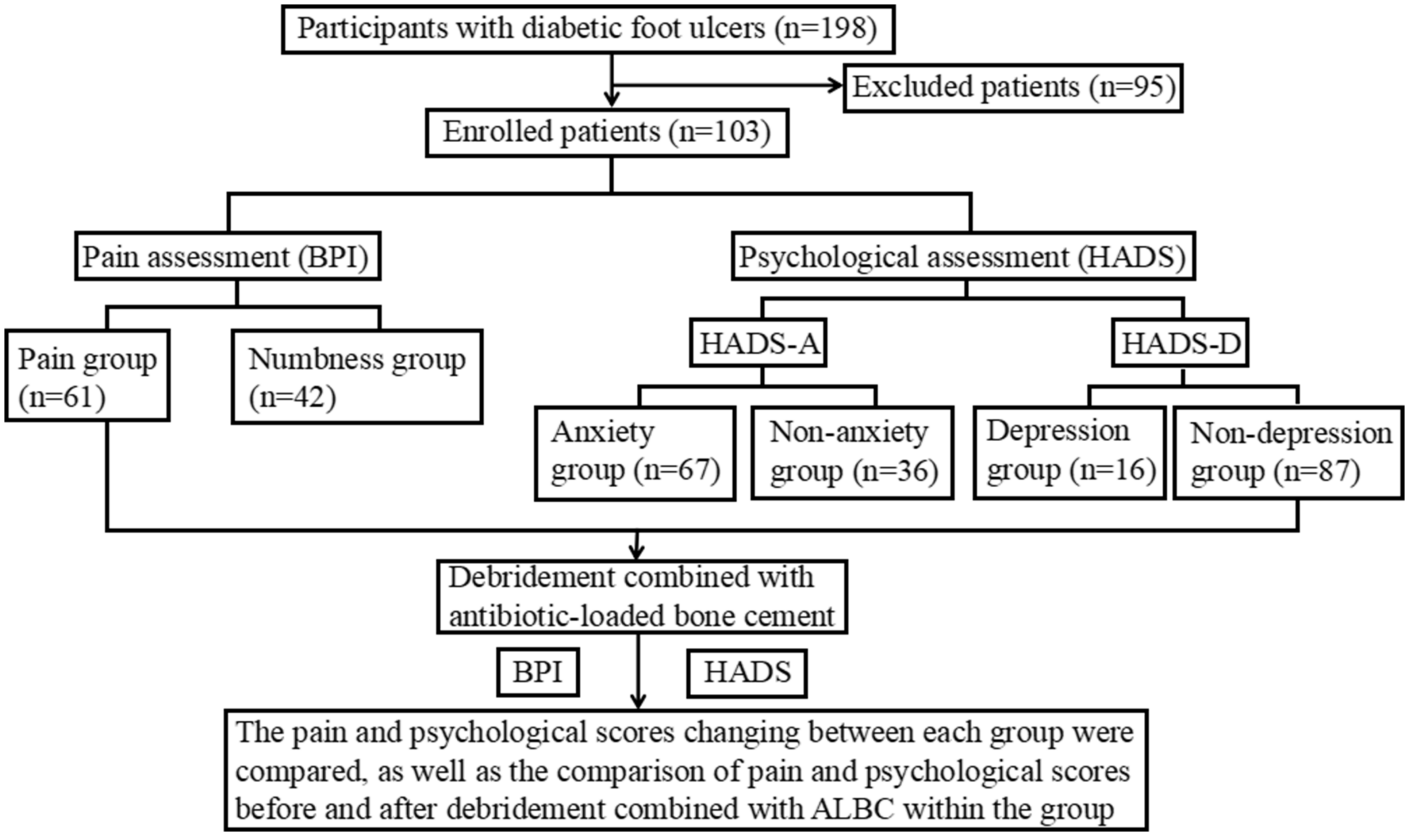
Flow chart of the study.

This study mainly adopts a self-controlled before-after design, and the research flowchart is presented in Figure 1. Based on the inclusion and exclusion criteria, a total of 103 DFU patients were enrolled, and relevant clinical data were collected. All patients received the treatment of debridement combined with ALBC, and the main components of ALBC are gentamicin and polymethylmethacrylate. Meanwhile, the BPI and HADS were used to assess pain levels and psychological status in DFU patients, respectively, that occurred 72 h before ALBC application and 72 h following ALBC removal, to investigate the effects of such treatment on pain and psychological well-being of DFU patients through a self-before-after comparison.

### 2.5 Statistical analysis

Statistical analysis was performed using SPSS (Statistical Product and Service Solutions) version 27.0. The comparisons between groups focused on painful and painless patients, anxious and non-anxious patients, and depressed and non-depressed patients. Descriptive statistics were used to report the baseline characteristics of all patients. Normally distributed continuous variables were presented as mean ± standard deviation, and comparisons between two groups were made using the *t*-test. Meanwhile, non-normally distributed data were expressed as medians and interquartile ranges, with the Wilcoxon test applied for group comparisons. To address potential confounding effects when comparing outcomes between groups based on baseline characteristics, we performed additional multiple linear regression analyzes for continuous outcome variables. Variables that showed significant differences between groups at baseline were included as covariates in these models to adjust for their effects. For the pain and psychological status within the same group of patients before and after debridement combined with ALBC, analyzes were conducted using either the signed-rank test or two-way ANOVA, depending on whether the data followed a normal distribution. Categorical variables were expressed as *n* (%), and comparisons were made using the χ^2^ test. Values of *p* < 0.05 were considered statistically significant.

## 3 Results

### 3.1 Baseline characteristics of participants

A total of 103 patients with DFUs were included in this study, and the baseline characteristics of all participants are summarized in Table 1. The average age of the patients was 56.5 years (ranging from 38 to 82 years), with 76 male patients (73.79%) and 27 female patients (26.21%). Among the participants, the proportion of smokers (20.39%) was slightly higher than that of drinkers (14.56%). Otherwise, most patients had a history of diabetes lasting 5–10 years, with the duration of DFUs ranging from 10 to 60 days. Meanwhile, the majority of patients underwent debridement combined with ALBC treatment for 4–6 weeks. Additionally, most patients with DFUs were identified with Wagner grade III (40.78%) or IV (48.54%), and 61 patients (59.22%) were reported experiencing pain.

**Table 1:** Baseline characteristics of all patients with DFUs.

Characteristics	All patients (*n* = 103)
Age (years)	56.5 ± 8.67
Gender	
Male	76 (73.79%)
Female	27 (26.21%)
Height (cm)	165.01 ± 7.38
Weight (kg)	62.98 ± 9.75
BMI (kg/m^2^)	23.29 ± 3.43
SBP (mmHg)	125.00 (112.00–137.00)
DBP (mmHg)	77.07 ± 11.67
Smoking	21 (20.39%)
Drinking	15 (14.56%)
Patients with pain	61 (59.22%)
Diabetes duration (years)	10.00 (5.00–10.00)
DFU duration (days)	23.00 (10.00–60.00)
ALBC duration (weeks)	5.00 (4.00–6.00)
FBG (mmol/L)	9.37 (7.26–13.41)
HbA1c (%)	10.20 ± 2.59
Ulcer site	
Left foot	41 (39.81%)
Right foot	55 (53.40%)
Both feet	7 (6.80%)
Wagner classification	
II	11 (10.68%)
III	42 (40.78%)
IV	50 (48.54%)
WBC (×10^9^/L)	9.16 (6.99–13.21)
RBC (×10^12^/L)	3.77 (3.17–4.23)
Hb (g/L)	108.00 (89.50–120.50)
Albumin (g/L)	32.71 ± 5.87
Globulin (g/L)	30.00 (27.00–34.00)
A/G^a^	1.00 (0.90–1.40)
Prealbumin (mg/L)	136.00 (73.00–185.00)
Ccr (mL/min)	78.30 (66.30–108.18)
Urea (mmol/L)	5.70 (4.10–7.70)
Creatinine (μmol/L)	80.00 (58.00–101.00)

BMI, body mass index; SBP, systolic blood pressure; DBP, diastolic blood pressure; DFU, diabetic foot ulcer; ALBC, antibiotic-loaded bone cement; FBG, fasting blood glucose; HbA1c, glycated hemoglobin type A1c; WBC, white blood cell; RBC, red blood cell; Hb, hemoglobin; Ccr, creatinine clearance rate.

a A/G: Albumin/Globulin.

### 3.2 The relationship between the levels of pain, anxiety and depression in DFU patients and their demographic and clinical characteristics

The 103 DFU patients were divided into a pain group and a numbness group according to pain status. The baseline characteristics of DFU patients in the two groups are shown in Table 2. There were 61 patients in the pain group, with an average age of 56.74 ± 9.59 years. Meanwhile, there were 42 patients in the numbness group, with an average age of 56.14 ± 7.22 years. The mean BMI of the pain group was 23.85 ± 3.41 kg/m2, which was higher than that of the numbness group with a mean BMI of 22.41 ± 3.42 kg/m2 (*p* < 0.05), suggesting that pain in DFU patients was associated with higher BMI. In addition, the number of patients with ulcers on both feet in the numbness group was higher than that in the pain group (*p* < 0.05). At the same time, the proportion of patients with Wagner grade III in the pain group was higher than that in the numbness group, while the proportion of patients with Wagner grade IV in the numbness group was higher than that in the pain group (*p* < 0.05). In addition, albumin and hemoglobin levels were higher in the pain group than those in the numbness group (*p* < 0.05). However, there were no significant differences in age, gender, FBG, HbA1c, WBC, RBC, prealbumin, urea, and creatinine between the two groups (*p* > 0.05).

**Table 2:** Baseline characteristics of DFU patients in the pain group and numbness group.

Characteristics	Pain group (*n* = 61)	Numbness group (*n* = 42)	*P*
Age (years)	56.74 ± 9.59	56.14 ± 7.22	0.721
Gender			0.652
Male	46 (75.41%)	30 (71.43%)	
Female	15 (24.59%)	12 (28.57%)	
Height (cm)	167.00 (160.00–170.00)	163.00 (160.00–170.00)	0.318
Weight (kg)	64.50 ± 10.00	60.96 ± 9.14	0.075
BMI (kg/m^2^)	23.85 ± 3.41	22.41 ± 3.42	**0.045**
SBP (mmHg)	128.00 (109.50–138.50)	124.00 (112.75–128.25)	0.213
DBP (mmHg)	78.67 ± 13.02	74.74 ± 9.02	0.073
Smoking	14 (22.95%)	7 (16.67%)	0.437
Drinking	11 (18.03%)	4 (9.52%)	0.229
Diabetes duration (years)	10.0 (3.0–10.0)	7.50 (3.5–10.0)	0.451
DFU duration (days)	30.0 (10.0–60.0)	25.0 (10.0–52.5)	0.409
ALBC duration (weeks)	5.0 (4.0–6.0)	4.0 (4.0–5.0)	0.337
FBG (mmol/L)	9.31 (7.20–12.92)	10.11 (7.25–15.91)	0.172
HbA1c (%)	10.20 (7.70–11.90)	10.65 (8.78–12.28)	0.257
Ulcer site			
Left foot	27 (44.26%)	14 (33.33%)	0.265
Right foot	33 (54.10%)	22 (52.38%)	0.864
Both feet	1 (1.64%)	6 (14.29%)	**0.035**
Wagner classification			
II	7 (11.48%)	4 (9.52%)	1.000
III	30 (49.18%)	12 (28.57%)	**0.036**
IV	24 (39.34%)	26 (61.90%)	**0.024**
WBC (×10^9^/L)	9.63 (7.20–13.84)	8.58 (6.78–12.35)	0.550
RBC (×10^12^/L)	3.78 (3.34–4.44)	3.67 (2.89–4.15)	0.156
Hb (g/L)	110.50 (95.25–127.00)	103.50 (80.00–115.25)	**0.028**
Albumin (g/L)	33.35 (29.43–38.35)	30.95 (27.90–35.18)	**0.040**
Globulin (g/L)	30.00 (26.00–35.00)	30.00 (27.00–33.00)	0.806
A/G^a^	1.10 (0.90–1.48)	1.00 (0.90–1.30)	0.242
Prealbumin (mg/L)	164.00 (80.75–225.25)	112.50 (68.75–176.50)	0.164
Ccr (mL/min)	79.57 (68.94–105.62)	75.82 (55.72–112.70)	0.724
Urea (mmol/L)	6.10 (4.60–8.50)	4.75 (3.78–7.05)	0.179
Creatinine (μmol/L)	80.00 (61.50–100.00)	78.50 (54.50–102.25)	0.723

Significant *p*-values (*p* < 0.05) are presented in bold. BMI, body mass index; SBP, systolic blood pressure; DBP, diastolic blood pressure; DFU, diabetic foot ulcer; ALBC, antibiotic-loaded bone cement; FBG, fasting blood glucose; HbA1c, glycated hemoglobin type A1c; WBC, white blood cell; RBC, red blood cell; Hb, hemoglobin; Ccr, creatinine clearance rate.

a A/G: Albumin/Globulin.

Among 103 patients with DFUs, those with HDS-A scores ranging from 0 to 7 were classified into the non-anxiety group, while those with scores between 8 and 21 were classified into the anxiety group. Similarly, those patients with HDS-D scores between 0 and 7 were classified as the non-depression group, and those with scores between 8 and 21 were classified as the depression group. The baseline characteristics of the DFU patients divided into these groups according to different criteria are shown in Tables 3, 4, respectively. Among the participants, 67 patients were classified into the anxiety group and 36 into the non-anxiety group. The proportion of patients experiencing numbness was slightly higher in the anxiety group than that in the non-anxiety group (*p* = 0.049), and the albumin levels in the anxiety group were significantly lower than those in the non-anxiety group (*p* = 0.008). Furthermore, depression scores were significantly higher in the anxiety group compared to the non-anxiety group (*p* < 0.001). However, no significant differences were found between the two groups in terms of age, gender, pain severity, pain-related impact, FBG, HbA1c, and other aspects. Additionally, there were 16 patients in the depression group and 87 patients in the non-depression group. Depressed patients had significantly higher pain severity and pain-related impact scores, as well as higher anxiety scores compared to non-depressed patients (*p* < 0.001). However, no statistical differences occurred between groups regarding diabetes-related characteristics or other laboratory indicators related to blood routine, liver function, and renal function.

**Table 3:** Baseline characteristics of DFU patients in the anxiety group and non-anxiety group.

Characteristics	HADS-A score <8 (*n* = 36)	HADS-A score ≥8 (*n* = 67)	*P*
Age (years)	57.833 ± 8.399	55.776 ± 8.789	0.253
Gender			0.837
Male	27 (75.00%)	49 (73.13%)	
Female	9 (25.00%)	18 (26.87%)	
Height (cm)	63.79 ± 10.81	63.05 ± 8.87	0.674
Weight (kg)	163.82 ± 7.53	165.64 ± 7.33	0.242
BMI (kg/m^2^)	23.75 ± 3.66	23.02 ± 3.29	0.327
SBP (mmHg)	127.5 (108.75–143.5)	125.0 (112.0–135.0)	0.376
DBP (mmHg)	79.0 (65.0–88.25)	74.0 (68.0–85.0)	0.497
Smoking	9 (25.00%)	12 (17.91%)	0.394
Drinking	4 (11.11%)	11 (16.42%)	0.467
Diabetes duration (years)	9.0 (4.5–10.0)	10.0 (5.0–15.0)	0.594
DFU duration (days)	15.0 (7.0–30.0)	30.0 (10.0–60.0)	0.391
ALBC duration (weeks)	5.0 (4.0–6.0)	5.0 (4.0–6.0)	0.550
Patients with pain	26 (72.22%)	35 (52.24%)	**0.049**
Pain severity	15.0 (0.0–22.75)	13.0 (0.0–28.0)	0.641
Pain-related impacts	19.0 (0.0–25.75)	26.0 (0.0–40.0)	0.223
FBG (mmol/L)	11.19 (6.76–15.8)	10.41 (7.39–14.22)	0.676
HbA1c (%)	9.80 ± 2.69	10.42 ± 2.53	0.282
Ulcer site			
Left foot	17 (47.22%)	24 (35.82%)	0.260
Right foot	18 (50.00%)	37 (55.22%)	0.612
Both feet	1 (2.78%)	6 (8.96%)	0.437
Wagner classification			
II	3 (8.33%)	8 (11.94%)	0.818
III	15 (41.67%)	27 (40.30%)	0.893
IV	18 (50.00%)	32 (47.76%)	0.828
WBC (×10^9^/L)	8.53 (6.13–11.87)	9.39 (7.25–13.60)	0.449
RBC (×10^12^/L)	3.79 (3.50–4.27)	3.69 (3.03–4.20)	0.754
Hb (g/L)	114.0 (97.50–125.50)	107.50 (88.25–120.00)	0.507
Albumin (g/L)	32.8 (31.23–37.45)	30.70 (27.50–35.08)	**0.008**
Globulin (g/L)	28.50 (25.75–34.00)	30.00 (27.00–35.00)	0.679
A/G^a^	1.10 (1.00–1.50)	1.00 (0.80–1.38)	0.080
Prealbumin (mg/L)	162.00 (93.50–226.00)	96.00 (64.25–184.75)	0.106
Ccr (mL/min)	5.85 (4.58–7.78)	5.70 (4.10–7.80)	0.252
Urea (mmol/L)	86.50 (65.00–104.25)	80.00 (61.25–101.00)	0.415
Creatinine (μmol/L)	74.01 (67.07–103.46)	87.76 (60.71–111.30)	0.193
HADS-D score	3.0 (2.0–3.0)	6.0 (5.0–7.0)	**<0.001**

Significant *p*-values (*p* < 0.05) are presented in bold. BMI, body mass index; SBP, systolic blood pressure; DBP, diastolic blood pressure; DFU, diabetic foot ulcer; ALBC, antibiotic-loaded bone cement; FBG, fasting blood glucose; HbA1c, glycated hemoglobin type A1c; WBC, white blood cell; RBC, red blood cell; Hb, hemoglobin; Ccr, creatinine clearance rate; HADS-D, hospital anxiety depression scale-depression.

a A/G: Albumin/Globulin.

**Table 4:** Baseline characteristics of DFU patients in the depression group and non-depression group.

Characteristics	HADS-D score <8 (*n* = 87)	HADS-D score ≥8 (*n* = 16)	*P*
Age (years)	57.489 ± 9.0142	58.667 ± 10.1119	0.260
Gender			0.668
Male	63 (72.41%)	13 (81.25%)	
Female	24 (27.59%)	3 (18.75%)	
Height (cm)	62.89 ± 10.07	63.0 ± 8.01	0.405
Weight (kg)	165.32 ± 8.27	162.22 ± 6.53	0.964
BMI (kg/m^2^)	23.04 ± 3.55	24.09 ± 4.12	0.579
SBP (mmHg)	123.0 (112.0–138.0)	128.0 (109.5–135.5)	0.477
DBP (mmHg)	75.66 ± 11.15	76.67 ± 12.47	0.747
Smoking	17 (19.54%)	4 (25.00%)	0.872
Drinking	12 (13.79%)	3 (18.75%)	0.896
Diabetes duration (years)	10.0 (5.0–15.0)	10.0 (4.0–14.5)	0.071
DFU duration (days)	20.0 (7.0–30.0)	60.0 (14.0–212.5)	0.056
ALBC duration (weeks)	5.0 (4.0–6.0)	4.0 (3.0–6.0)	0.832
Patients with pain	49 (56.32%)	12 (75.00%)	0.162
Pain severity	7.0 (0.0–23.0)	29.0 (0.0–31.0)	**<0.001**
Pain-related impacts	10.0 (0.0–30.0)	43.0 (0.0–44.0)	**<0.001**
FBG (mmol/L)	10.48 (7.61–13.33)	8.95 (7.05–12.44)	0.435
HbA1c (%)	10.02 ± 2.40	10.80 ± 3.41	0.186
Ulcer site			
Left foot	33 (37.93%)	8 (50.0%)	0.365
Right foot	47 (54.02%)	8 (50.0%)	0.767
Both feet	7 (8.05%)	0	0.526
Wagner classification			
II	9 (10.34%)	2 (12.50%)	1.000
III	37 (42.53%)	5 (31.25%)	0.399
IV	41 (47.13%)	9 (56.25%)	0.502
WBC (×10^9^/L)	9.61 (7.33–14.36)	11.72 (7.03–17.87)	0.934
RBC (×10^12^/L)	3.79 (3.07–4.21)	3.95 (3.16–4.29)	0.981
Hb (g/L)	109.0 (89.0–127.0)	105.0 (91.5–123.0)	0.911
Albumin (g/L)	32.19 ± 6.46	30.64 ± 6.95	0.208
Globulin (g/L)	30.38 ± 5.89	31.33 ± 4.36	0.877
A/G^a^	1.0 (0.9–1.3)	0.9 (0.75–1.3)	0.164
Prealbumin (mg/L)	95.0 (65.0–176.0)	167.0 (64.5–190.0)	0.571
Ccr (mL/min)	6.0 (4.6–8.0)	6.1 (3.7–9.15)	0.516
Urea (mmol/L)	88.0 (62.0–108.0)	62.0 (58.0–87.0)	0.418
Creatinine (μmol/L)	76.67 ± 29.28	93.02 ± 28.93	0.475
HADS-A score	8.0 (6.0–11.0)	15.0 (13.0–15.75)	**<0.001**

Significant *p*-values (*p* < 0.05) are presented in bold. BMI, body mass index; SBP, systolic blood pressure; DBP, diastolic blood pressure; DFU, diabetic foot ulcer; ALBC, antibiotic-loaded bone cement; FBG, fasting blood glucose; HbA1c, glycated hemoglobin type A1c; WBC, white blood cell; RBC, red blood cell; Hb, hemoglobin; Ccr, creatinine clearance rate; HADS-A, hospital anxiety depression scale-anxiety.

a A/G: Albumin/Globulin.

### 3.3 Multiple linear regression analyzes adjusting for baseline confounders

To address potential confounding effects from baseline imbalances, multiple linear regression analyzes were conducted to identify independent factors associated with improvements in pain, anxiety, and depression scores after treatment. The results are summarized in Table 5.

**Table 5:** Multiple regression analysis in factors associated with improvements in anxiety, depression, and pain scores after treatment.

Items	Variables	*β*	*P*
Improvement in pain score	Patients with pain	0.657	**0.015**
	BMI	−0.028	0.457
	Hb	<0.001	0.835
	Albumin	−0.017	0.433
	Ulcerated foot^a^		
	Right foot	−0.100	0.838
	Left foot	0.041	0.936
	Wagner classification^b^		
	III	0.412	0.364
	IV	0.581	0.193
Improvement in anxiety score	Patients with anxiety	1.811	**<0.001**
	Patients with pain	0.463	0.069
	Albumin	0.015	0.474
	HADS-D	0.113	0.114
Improvement in depression score	Patients with depression	0.958	**0.004**
	Pain severity	−0.007	0.754
	Pain-related impacts	0.005	0.775
	HADS-A	0.124	**<0.001**

Significant *p*-values (*p* < 0.05) are presented in bold. BMI, body mass index; Hb, hemoglobin; HADS-A, hospital anxiety depression scale-anxiety; HADS-D, hospital anxiety depression scale-depression.

a Reference: Both feet.

b Reference: Wagner II.

After adjusting for BMI, hemoglobin, albumin, ulcer site, and Wagner classification, baseline pain status was a significant independent predictor of greater improvement in pain scores. Patients who reported pain at baseline had a significantly greater reduction in BPI scores after treatment compared to those with numbness (*β* = 0.657, *p* = 0.015).

Similarly, for psychological outcomes, baseline psychological status was a strong independent predictor of its respective improvement. Patients with baseline anxiety showed a significantly greater reduction in HADS-A scores after adjusting for baseline pain status, albumin, and depression scores (*β* = 1.811, *p* < 0.001). Likewise, patients with baseline depression experienced a significantly greater improvement in HADS-D scores after controlling baseline pain severity, pain-related impacts, and anxiety scores (*β* = 0.958, *p* = 0.004). Furthermore, a higher baseline anxiety score was independently associated with greater improvement in depression scores (*β* = 0.124, *p* < 0.001). Other variables included in the models were not significantly associated with the outcomes in these adjusted analyzes.

### 3.4 Effect of debridement combined with ALBC on pain and psychological status in DFU patients

Table 6 presents the BPI scores of pain intensity and pain-related items of all DFU patients treated with debridement combined with ALBC. The pain severity and pain-related scores for all DFU patients showed a non-normal distribution. After debridement combined with ALBC, the total scores for both pain severity and its related impacts decreased significantly in all participants and the same as the pain group. However, most painless patients before debridement combined with ALBC treatment did not experience an obvious change in pain status after treating with debridement combined with ALBC. Therefore, the median, 25th percentile, and 75th percentile of these item scores concerning pain severity and related impacts for patients in the numbness group were all 0.

**Table 6:** The characteristics of pain status in DFU patients by the pain-related numerical scoring items of BPI.

BPI items	Group	Before treatment	After treatment	*Z*	*P*
Pain severity	All patients (*n* = 103)	15.0 (0–25.0)	15.0 (0–24.0)	−3.302	**0.001**
	Pain group (*n* = 61)	23.0 (16.0–29.0)	23.0 (17.0–27.0)	−3.302	**0.001**
	Numbness group (*n* = 42)	0.0 (0.0–0.0)	0.0 (0.0–0.0)	0	1.000
Pain-related impacts	All patients (*n* = 103)	22.0 (0–34.0)	20.0 (0–32.0)	−5.456	**<0.001**
	Pain group (*n* = 61)	32.0 (24.5–41.5)	30.0 (21.5–37.5)	−5.456	**<0.001**
	Numbness group (*n* = 42)	0.0 (0.0–0.0)	0.0 (0.0–0.0)	0	1.000

Significant *p*-values (*p* < 0.05) are presented in bold.

Table 7 shows the HADS scores of anxiety and depression levels of all DFU patients treated with debridement combined with ALBC. Compared with the patients before debridement combined with ALBC, the anxiety and depression scores of those after debridement combined with ALBC were significantly decreased (*p* < 0.001). Moreover, after debridement combined with ALBC, the number of patients without anxiety and depression increased, the number of patients with moderate and severe anxiety decreased, and the number of patients with moderate depression also decreased (*p* < 0.05).

**Table 7:** The characteristics of psychological status in DFU patients by the anxiety and depression-related numerical scoring items of HADS.

Outcome	Before debridement combined with ALBC (*n* = 1.00003)	After debridement combined with ALBC (*n* = 103)	*P*
Anxiety primary outcome			
HADS-A	10.0 (6.0–12.0)	7.0 (5.0–10.0)	<**0.001**
Severity			
No symptoms (0–7)	36 (34.95%)	53 (51.46%)	**0.017**
Mild symptoms (8–10)	22 (21.36%)	31 (30.10%)	0.151
Moderate symptoms (11–14)	36 (34.95%)	19 (18.45%)	**0.007**
Severe symptoms (>15)	9 (8.74%)	0	**0.006**
Depression primary outcome			
HADS-D	5.0 (3.0–6.0)	4.0 (3.0–6.0)	<**0.001**
Severity			
No symptoms (0–7)	87 (84.47%)	99 (96.12%)	**0.005**
Mild symptoms (8–10)	15 (14.56%)	4 (3.88%)	**0.008**
Moderate symptoms (11–14)	1 (0.97%)	0	1.000*
Severe symptoms (>15)	0	0	1.000

* Fisher exact test. Significant *p*-values (*p* < 0.05) are presented in bold. ALBC, antibiotic-loaded bone cement; HADS-A, hospital anxiety depression scale-anxiety; HADS-D, hospital anxiety depression scale-depression.

To further explore whether different pain states affect the psychological status of DFU patients, we compared the total scores of pain severity, pain-related impacts, and levels of anxiety and depression between patients reporting pain and those experiencing numbness, as shown in Table 8. We found that, prior to debridement combined with ALBC, patients in the pain group had significantly higher scores for both pain severity and its related impacts compared to those in the numbness group (*p* < 0.001). This significant difference persisted after debridement combined with ALBC (*p* < 0.001). However, there were no significant differences in anxiety and depression levels between the two groups, either before or after debridement combined with ALBC (*p* > 0.05).

**Table 8:** The characteristics of pain levels and psychological status in DFU patients with numbness compared with those with pain.

Outcome	Time	Pain group (*n* = 61)	Numbness group (*n* = 42)	Z	*P*
Pain severity	Before debridement combined with ALBC	23.0 (16.0–29.0)	0.0 (0.0–0.0)	−8.909	**<0.001**
	After debridement combined with ALBC	23.0 (17.0–27.0)	0.0 (0.0–0.0)	−8.907	**<0.001**
Pain-related impacts	Before debridement combined with ALBC	32.0 (24.5–41.5)	0.0 (0.0–0.0)	−8.906	**<0.001**
	After debridement combined with ALBC	30.0 (21.5–37.5)	0.0 (0.0–0.0)	−8.905	**<0.001**
Anxiety	Before debridement combined with ALBC	8.0 (5.0–12.25)	10.0 (7.5–12.0)	−0.050	0.960
	After debridement combined with ALBC	7.0 (4.0–9.25)	7.5 (6.0–10.0)	−0.581	0.562
Depression	Before debridement combined with ALBC	4.0 (3.0–6.25)	5.0 (3.0–6.0)	−0.085	0.932
	After debridement combined with ALBC	4.0 (3.0–6.0)	4.0 (3.0–5.25)	−0.229	0.819

Significant *p*-values (*p* < 0.05) are presented in bold. ALBC, antibiotic-loaded bone cement.

### 3.5 Wound assessment after debridement combined with ALBC

To investigate the correlation between pain levels, psychological status, and wound condition after undergoing debridement combined with ALBC treatment in patients with DFUs, wound assessment was used from several aspects, including surrounding skin condition, wound area, exudate, and tissue repair. As shown in Table 9, more than half of the patients showed no redness or swelling around the wound (*n* = 52), and over 70% of the patients had no increase in wound area (*n* = 80). Meanwhile, only a small number of patients (*n* = 30) exhibited yellow purulent exudate. After the removal of ALBC, we observed that 69.9% of the patients’ wounds had progressed to the healing stage, including the formation of induced membranes and the growth of granulation tissue.

**Table 9:** Wound condition of all DFU patients after debridement combined with ALBC.

Characteristics	All patients (*n* = 103)
Surrounding skin	
Red and swollen	42 (40.78%)
Black	9 (8.74%)
Normal	52 (50.49%)
Wound area	
Reduce	30 (29.13%)
Unchange	50 (48.54%)
Increase	23 (22.33%)
Exudate	
No obvious exudate	21 (20.39%)
A little exudate	52 (50.49%)
Purulent exudate	30 (29.13%)
Tissue repair	
Induced membrane	34 (33.01%)
New granulation tissue	38 (36.89%)
Inflammatory granulation tissue	31 (30.10%)

## 4 Discussion

DFUs are among the most common complications in patients with diabetes, which not only impairs physical health but also produces a significant impact on mental well-being. That’s because the presence of DFUs is often accompanied by long-term infection, pain, and mobility limitations, resulting in a significant decline in the quality of life of patients (14). Meanwhile, as the number of patients with such chronic diseases like DFUs continues to rise, the “patient-centered” healthcare approach has gained wide attention for the public (15). This approach not only focuses on the diagnosis and treatment of diseases but also emphasizes patients’ multidimensional aspects such as psychological health and quality of life. Pain management and psychological interventions for diseases are aligned with this trend, playing an essential role in improving long-term outcomes for patients. Currently, the treatment of DFUs is primarily centered around infection control and wound healing, with patients’ subjective experiences often overlooked. Therefore, this study explores the application of debridement combined with ALBC in DFU patients, particularly its role in alleviating pain and improving psychological health. The results indicate a potential benefit of debridement combined with ALBC for infection control, as well as for reducing pain and alleviating symptoms of anxiety and depression, providing new insights into the comprehensive treatment of DFUs.

### 4.1 Pain in DFUs

Most of the 103 patients with DFUs enrolled in this study were middle-aged and elderly men, and their course of diabetes mellitus was about 5-10 years long. Among them, 61 patients reported obvious pain, while the other 42 patients experienced numbness, and their Wagner grades were mainly grade III and IV, suggesting that patients with severe lesions are usually accompanied by significant peripheral neuropathy. Notably, pain is usually caused by tissue damage or inflammatory stimulation, while numbness is mostly caused by nerve compression or injury. Therefore, diabetes-related immune disorders, especially neutrophil which amplifies and perpetuates tissue inflammation by providing cytokines and chemokines that promote the accumulation of pro-inflammatory leucocytes, could lead to microbial colonization in the deep ulcer wounds of feet, triggering an inflammatory response or tissue damage that in turn results in foot pain (16). In addition, microcirculation disorders and excessive oxidative stress in the peripheral nerves of the lower extremities are the key factors of peripheral neuropathy in DFUs (17). Therefore, microcirculation disorders caused by long-term hyperglycemia result in insufficient blood supply to the terminal nerves with ischemia, hypoxia and edema, which leads to the development of numbness. However, with nerve edema, the inner membrane of the wrapped nerve is compressed, which aggravates the nerve edema and forms a vicious circle. On the other hand, the nerve with edema passing through the pipeline between muscles, bones and fascia is compressed, which aggravates edema and forms a more serious vicious circle, eventually leading to the worse situation of foot numbness in DFU patients.

Patients with DFUs who report pain often have higher BMI, which is similar to the results of previous studies that found hyperglycemia can interfere with pain modulation, enhance pain sensitivity, and weaken pain inhibition in people with excess fat mass (18, 19). Meanwhile, obesity increases the burden on the feet, which may make diabetic patients more susceptible to foot ulcers. Due to the pain associated with these ulcers, daily activity is significantly reduced, exacerbating obesity and creating a positive feedback loop of “obesity-pain-reduced activity-metabolic deterioration”. Furthermore, emerging research implicates the neuroimmune response, including the recruitment and activation of macrophages, in the pathogenesis of diabetic neuropathy, potentially influencing the symptoms like pain and numbness observed in our patient groups (20). This evolving understanding may inform future therapeutic strategies targeting immune modulation in addition to infection control (21). Moreover, a higher proportion of patients in the pain group are classified as Wagner grade III, while more patients in the numbness group are classified as grade IV. Patients with Wagner grade III have severe infections of deep ulcer wounds with extensive tissue damage or inflammatory response which are more likely to trigger pain. In contrast, patients with Wagner grade IV are present with localized gangrene and bone defects, and due to inadequate blood supply, they are more prone to nerve edema, leading to numbness. Additionally, patients with numbness experience significant reductions in pain, temperature, and tactile sensation, making them less sensitive to the change of foot pain or temperature, thereby increasing the risk of bilateral foot ulcers. These findings suggest that pain perception disorders may be linked to the severity of obesity and the progression of DFUs, with numbness being a significant risk factor for the deterioration of DFUs. Additionally, patients in the numbness group had almost no pain-related scores, and debridement combined with ALBC did not show significant improvements. This indicates that the onset of numbness suggests irreversible severity of peripheral neuropathy.

### 4.2 Psychological status in DFUs

With the continuous advancements in medical research and public health awareness, the focus of disease management has expanded beyond the physiological level. Increasingly, people are recognizing the crucial role that psychological factors play in the onset, progression, and prognosis of various diseases. This is particularly important in the context of DFUs, where understanding the psychological status of patients is essential. The treatment of DFUs is a long-term and complex process that involves ongoing blood glucose management, foot care, and wound treatment, among other aspects. In the face of these complex therapeutic measures, patients may experience psychological issues such as anxiety and depression. These emotional disorders often lead to decreased treatment adherence, which further affects the effectiveness of disease control (22). For example, Westby et al. (23) also found a correlation between depression and increased risk of diabetic foot ulcers through research. In this study, 67 DFU patients exhibited different levels of anxiety symptoms, while 36 DFU patients did not report anxiety. Meanwhile, 16 DFU patients reported different levels of depression, and 87 DFU patients showed no depressive symptoms, which suggests that emotional disorders are commonly present in DFU patients.

Anxiety is an emotional state that is typically triggered by perceived threats or dangers in the future, although the severity of anxiety may not always correlate with the actual event. On the other hand, depression often stems from a profound sense of helplessness and hopelessness in response to past and present failures or difficulties (24). In this study, the HADS-A and HADS-D scales were used to assess anxiety and depression in DFU patients. The results showed a significant positive correlation between the levels of anxiety and depression, suggesting that anxiety and depression are both independent yet coexisting conditions, potentially triggering each other (25). Chronic anxiety may lead to a sense of helplessness, which could develop into depression. Meanwhile, the low mood associated with depression may amplify worries about the future, creating a vicious cycle of anxiety. Furthermore, our study found that the severity of pain was more strongly correlated with depression than with anxiety, because pain scores in depressed patients were significantly higher than in those without depression, while no similar findings were observed in anxious patients. This suggests that an increase in pain severity is more likely to evoke a sense of helplessness about the present, rather than heightening concerns about the future. Additionally, the lower serum albumin levels in the numbness group, compared to the pain group, may be partly attributed to the higher proportion of anxious individuals in the numbness group. The mechanism behind this could be that anxiety triggers activation of the sympathetic-adrenal-medullary axis and the hypothalamic–pituitary–adrenal axis, leading to increased levels of cortisol which is a stress hormone regulating various physiological processes (26). Cortisol promotes protein breakdown and inhibits amino acid uptake in extrahepatic tissues, thereby reducing protein synthesis. At the same time, it accelerates the entry of amino acids into the liver to become raw material for gluconeogenesis, thus reducing the plasma albumin content (27). Moreover, numbness itself reduces the patient’s sensitivity to pain and diminishes awareness of their health status, which could worsen their condition and potentially exacerbate anxiety. This further creates a vicious cycle, with anxiety influencing both physiological and psychological states.

### 4.3 Role of debridement combined with ALBC in DFUs

This study found that following debridement combined with ALBC treatment, there was a significant reduction in the pain levels and related impacts in the pain group of DFU patients, along with a significant decrease in the number of patients with moderate to severe anxiety and mild depression. However, due to the limited number of participants in this study, only one patient among 163 had moderate depression, and there were even no patients with severe depression. Therefore, the effect of this treatment on alleviating moderate to severe depressive symptoms could not be observed. Since this therapy greatly improved the impact of pain on daily life, walking, and sleep, it suggests that these factors play a significant role in influencing the psychological status of patients. However, further investigation revealed that the presence or absence of pain did not significantly affect the patients’ anxiety or depressive symptoms. These findings may indicate a complex relationship between pain and the emotional states of anxiety and depression in DFU patients. The improvement in psychological health after debridement combined with ALBC treatment may not be solely dependent on pain relief. Other factors, such as disease perception and social support, may play important roles in the change of psychological states (28, 29). This analysis helps to uncover the multidimensional factors contributing to the improvement of mental health in DFU patients and provides a more comprehensive perspective for future interventions.

DFUs always fail to heal quickly with simple drug treatment, particularly when there are severe infections, extensive tissue damage, and exposure to tendons and bone structures. Therefore, timely and effective debridement is essential for promoting the healing of foot ulcer wounds. The identification and removal of necrotic tissue, as well as the preservation and protection of healthy tissue, are key factors in determining the effectiveness of debridement. Current dealings with DFUs primarily include debridement, debridement combined with vacuum sealing drainage (VSD), and debridement combined with ALBC. Simple debridement involves the removal of necrotic tissue, muscle, and some necrotic bone fragments from the ulcer, though it has limited efficacy in infection control and results in slow granulation tissue growth (30). Otherwise, VSD involves covering or filling skin and soft tissue defects with polyvinyl alcohol-hydrated sodium alginate foam dressing containing a drainage tube that is connected to a negative pressure source, followed by sealing it with a biological semi-permeable membrane to change to a closed wound. This treatment effectively reduces exudate, inhibits bacterial growth, and accelerates blood circulation to improve blood supply (31, 32). However, it is dependent on the use of negative pressure equipment, which may not be available in primary healthcare settings, and its material costs and device usage increase treatment expenses. Additionally, patients must carry the device, which may limit their daily activities, and issues such as tube blockages or air leaks can exacerbate infection. Moreover, ALBC, a kind of stable antibiotic delivery system made by mixing antibiotics into bone cement composed of polymethyl methacrylate, forms an induction membrane around the wound, promoting granulation tissue proliferation (33). It releases high concentrations of antibiotics locally, effectively controlling infection and reducing inflammatory factors such as prostaglandin E2, which can reduce pain caused by nerve stimulation (34). Meanwhile, bone cement can mold to the shape of the wound, providing effective coverage for 4–6 weeks, which reduces repetitive irritation with pain. After using ALBC, patients experience too little discomfort to return home for self-care, easing their financial and psychological burdens. This may help explain the conclusions of this study and support the clinical advantages of debridement combined with ALBC in the treatment of DFUs, making it a widely applicable treatment in clinical practice.

Finally, through the wound assessment post-treatment, we observed that the debridement combined with ALBC therapy promoted tissue repair in the majority of DFU patients (69.9%) through the formation of induced membrane and granulation tissue. The induced membrane is a biofilm that is usually formed when using surgical methods such as Masquelet technology, which was originally used for the treatment of bone defects. By placing a spacer (such as bone cement) in the defect area and then forming a layer of biofilm around it, promoting local angiogenesis and providing growth factors and cytokines, thereby contributing to bone regeneration and tissue repair (35, 36). Meanwhile, granulation tissue is a reactive hyperplasia phenomenon to injury, which is mainly composed of new capillaries, fibroblasts and various inflammatory cells (37, 38). It can grow rapidly and fill the holes or gaps formed by injury. Both of these promote the wound healing process of DFUs. At the same time, our wound assessment findings provide compelling clinical evidence supporting the proposed mechanisms. The observation that the majority of patients’ wounds showed no enlargement (77.67%), reduced exudate (70.88%), and progressed to healing (69.9%) directly corroborates the efficacy of ALBC in controlling infection and promoting tissue repair. This tangible improvement in the wound bed is critically important for understanding the observed reductions in pain and psychological distress. Reduced bacterial burden and inflammation directly alleviate nociceptive stimulation and tissue damage, leading to less pain (39). Concurrently, visibly witnessing one’s wound transition from a state of infection and stagnation to active healing (e.g., formation of granulation tissue) can have a profound positive psychological impact. It reduces the uncertainty and fear associated with a non-healing wound, fostering hope, enhancing perceived control over the disease, and thereby directly reducing anxiety and depressive symptoms (40). Thus, the objective wound improvements are not merely a parallel outcome but are likely a central mechanistic link between ALBC treatment and the dual benefit of pain reduction and psychological improvement.

In conclusion, the use of debridement combined with ALBC in DFU patients appears to provide benefits in terms of pain reduction and psychological improvement. Notwithstanding these promising findings, it is crucial to interpret our results in the context of the study’s limitations (the retrospective design, single-center nature, and relatively small sample size). Therefore, these findings just provide preliminary support for the wider use of debridement combined with ALBC in treating DFUs. However, further larger, prospective, randomized controlled trials are needed to confirm these findings and explore the long-term effects of debridement combined with ALBC on DFU healing and patient well-being. Future studies should also investigate a comprehensive treatment approach that includes not only wound care but also pain management and psychological interventions and so on to improve the overall quality of life for DFU patients.

### 4.4 Limitations

Although this study yielded some meaningful results, several limitations should be acknowledged. First, as a single-arm, pre-post interventional study, lack of the control group, we cannot definitively elucidate that the observed improvements in pain and psychological scores were solely and directly due to the debridement combined with ALBC treatment. Other factors, such as the natural passage of time, or the concomitant standard care all patients received (including glycemic control and wound dressing changes), may have contributed to the outcomes. Therefore, our findings just indicated a promising association between treatment and symptom improvement, rather than conclusive evidence of causation. Future randomized controlled trials (RCTs) with appropriate control groups (e.g., patients receiving standard debridement and care without ALBC) are essential to confirm the efficacy of this treatment modality. Second, the post-hoc grouping of patients based on baseline symptoms (e.g., pain vs. numbness, anxiety vs. non-anxiety) for comparative analysis is a limitation, as it may introduce selection bias and confounding. Although we attempted to adjust for key confounding variables using regression models, residual confounding by unmeasured factors cannot be ruled out. The findings from these subgroup comparisons should be interpreted with caution. Third, the sample size in this study was relatively small, particularly in subgroups like the depression cohort (*n* = 16), which may lead to biases in the demographic characteristics, clinical features, and pain and psychological assessments across different subgroups. Fourthly, to enhance the accuracy and credibility of assessment, pain and psychological conditions in patients should be assessed using multiple clinical scales simultaneously. Furthermore, this study employed a single-center design, which may limit the external validity of the results due to regional and population differences. Thus, future research should include multicenter, large sample, prospective clinical trials to validate the effectiveness and safety of debridement combined with ALBC treatment on a larger scale. These are areas that need to be further addressed in future studies to better inform clinical practice.

## Data Availability

The original contributions presented in the study are included in the article/supplementary material, further inquiries can be directed to the corresponding authors.
